# Editorial: Advances in neuroimaging, genetic, and stimulation methods for cognitive and behavioral function investigation

**DOI:** 10.3389/fnhum.2026.2000021

**Published:** 2026-09-25

**Authors:** Haoran Yang, Xiaoluan Xia, Zhen Yuan

**Affiliations:** 1 Centre for Cognitive and Brain Sciences, University of Macau, Taipa, Macau SAR, China; 2 Faculty of Medicine, University of Macau, Taipa, Macau SAR, China; 3 Zhuhai UM Science and Technology Research Institute, Zhuhai, China

**Keywords:** brain imaging, brain stimulation, clinical translation, cognitive function, network analysis

Understanding cognitive and behavioral function increasingly requires linking distributed neural activity with physiological, clinical, and system-level processes. Advances in neuroimaging, genetic and epigenetic approaches, and non-invasive brain stimulation, alongside quantitative modeling and electrophysiology, now permit these relationships to be examined across spatial, temporal, and biological levels. This Research Topic brings eight contributions employing task-based fMRI, EEG, fNIRS, structural MRI, graph-theoretical analysis, clinical modeling, and high-definition transcranial direct current stimulation (HD-tDCS). Together they span executive function, anxiety, motor impairment, neurodegenerative disease, and cognitive risk, and include a systematic review of the epigenetic and psychoneuroimmune-endocrine mechanisms underlying implicit processes. Rather than isolated methodological developments, these contributions converge on three interlocking foci: the network-based characterization of cognitive architecture, the translational validation of clinical measurements, and the integration of intervention safety with systems-level conceptual frameworks. Collectively, they trace a validation pathway from network-level mechanisms to clinical utility and, ultimately, to practical boundaries of intervention—illustrating both the methodological expansion of neuroscience and the translational rigor it now demands.

## Network-based characterization of cognitive architecture

A key methodological shift in this Research Topic is from region-centered signal mapping toward network-based accounts of cognitive and behavioral function. Using task-based fMRI and graph analysis, Davis examined four subdomains of executive function and identified the right medial prefrontal cortex and bilateral precuneus as hub regions common to all four, suggesting a domain-general regulatory role. Distinct hubs additionally emerged in cognitive inhibition and mental shifting tasks, indicating flexible, task-specific network configurations; the author proposed that such a decentralized yet structured network may promote cognitive resilience. Using a similar network approach with resting-state EEG, Pavlova et al. found that higher math anxiety was associated with lower mean eigenvector centrality in alpha and theta bands, although associations were sensitive to threshold selection. Collectively, these studies demonstrate how graph-based analysis can characterize shared cognitive architecture and individual behavioral differences, while underscoring that network findings remain sensitive to analytic choices.

## Translational validation of clinical measurements

If network characterization addresses how cognitive architecture is organized, this second group of studies addresses whether measurements can reliably support clinical judgment—advancing a validation chain across feasibility, diagnostic modeling, longitudinal monitoring, and mechanistic pathway analysis. Debertshäeuser et al. conducted a feasibility study in nine participants performing n-back tasks before and after cycling, reporting satisfactory procedural and signal quality; load-dependent behavioral and hemodynamic responses provided preliminary evidence of task sensitivity, although the study was not designed to determine the effects of exercise intensity on cognition. Liu M. et al. developed a nomogram for upper-limb motor dysfunction severity in 275 ischemic stroke patients; despite good discriminative ability in internal validation, limited sensitivity and no association with activities of daily living currently restrict its utility to a supplementary assessment reference. He and Qiu addressed the longitudinal dimension in 37 patients with sporadic Creutzfeldt-Jakob disease, detecting periodic sharp wave complexes, often early after onset; a longitudinally followed case showed parallel EEG and MRI progression, although early EEG features were not associated with survival—findings that establish the early diagnostic value of EEG while delineating the limits of its prognostic utility. Liu C. et al. focused on cerebral small vessel disease, quantifying the volume of basal ganglia enlarged perivascular spaces (BG-EPVS) in 244 patients and identifying elevated HbA1c, greater BG-EPVS volume, and hypertension as independent risk factors for mild cognitive impairment, with BG-EPVS partially mediating the HbA1c–cognition association; however, this mediation was not reproduced after stratification by diabetes status, and causality cannot be established. Together, these four studies delineate the validation pathway that quantitative neuroimaging markers must traverse from bench to clinical decision-making, with each step accompanied by explicit acknowledgment of its methodological and interpretive limits.

## Intervention safety and systems-level integration

Methodological expansion occurs not only at observational and diagnostic levels but also at the level of intervention and conceptual integration. Carther-Krone and Ko systematically reviewed adverse event reporting for HD-tDCS in healthy and clinical populations, finding that adverse events within commonly used stimulation ranges were predominantly mild, transient, and localized. Severe reactions and dropout due to discomfort were uncommon, with no significant differences across the stimulation parameters. However, heterogeneity in assessment methods and reporting practices limits cross-study comparison, leading the authors to advocate for standardized tolerability metrics and complete reporting of both the presence and absence of adverse events. This work provides a methodological benchmark for safety evidence in neuromodulation research. Beyond intervention safety, Mundo et al. broadened the perspective to a multilevel biological integration, systematically examining PINE regulation, epigenetic mechanisms, and morphogenetic organization in relation to implicit processes. Their review supports associations between PINE dysregulation and stress-adaptive outcomes, and between environmental exposures and epigenetic modifications relevant to neurodevelopment; by contrast, morphogenetic principles lack direct quantitative support and are better regarded as a hypothesis-generating framework. By distinguishing empirical support from conceptual extension, this review extends the Research Topic toward molecular, physiological, and developmental integration while identifying hypotheses for prospective validation.

## Conclusion

Eight studies, three foci, one overarching trajectory: research on the neural mechanisms of cognitive and behavioral function is moving from single-method and single-modality observation toward multimethod, multimodal, and multiscale integration. Network-based characterization reveals shared hubs and flexible configurations within cognitive architecture; translational validation tests the reliability of measurement tools across feasibility, diagnosis, and mechanistic modeling; and intervention safety and system-level integration define the boundaries of application and conceptual inference. Each focus stands independently, yet all converge on a common direction: establishing sustainable validation pathways between methodological rigor and clinical utility.

